# Exploring the Implementation of Multiple Telementoring ECHO Programs From an Institutional and Organizational Perspective: Qualitative Study

**DOI:** 10.2196/75844

**Published:** 2025-11-13

**Authors:** M Gabrielle Pagé, Élise Develay, Annie Talbot, Rania Khemiri, Claire Wartelle-Bladou

**Affiliations:** 1 Department of Anesthesiology and Pain Medicine, Faculty of Medicine, Université de Montréal, 850 St-Denis, Office S03-910, Montreal, QC, H2X 0A9, Canada, 1 5148908000 ext 31601; 2 Research Center, Centre Hospitalier de l’Université de Montréal, Montreal, QC, Canada

**Keywords:** Extension for Community Healthcare Outcomes, ECHO, qualitative, implementation, hepatitis C, chronic pain, concomitant disorders, sustainability, barriers, facilitators

## Abstract

**Background:**

Project Extension for Community Healthcare Outcomes (ECHO) is an innovative model to increase capacity to treat patients in their community. Despite a growing body of evidence supporting its effectiveness, little is known about the implementation processes of multiple ECHO programs within an institution from the perspective of executives and institutional leaders.

**Objective:**

The study objective was to explore from an institutional and organizational standpoint the systemic characteristics that influence the implementation of Project ECHO programs, their growth within an ecosystem, and their sustainability.

**Methods:**

Focus groups and individual interviews were carried out with executives and leaders from an institution that implemented 3 Project ECHO programs, and verbatim were analyzed based on organizational readiness and implementation tools for Project ECHO.

**Results:**

This study highlighted the rarely reported perspectives of executives and institutional partners, shedding light on the organizational components that are essential to the deployment and sustainability of Project ECHO. Results reflect the intricate balance between institutional resources and its broader mission within a provincial, public health care system. In terms of acceptability, the fit between the projects and the institution’s values of innovation, contribution to the broader community, and improving patient trajectory was central from the organizational leaders’ standpoint. The structure of the projects and their rapid growth within the institution confirmed the adequacy with the institution. The projects benefited from temporary funds initially, and the lack of performance indicators that were easily measurable and the lack of recognition for invested time from clinicians were barriers to moving toward sustainability. Organizational characteristics, including a decentralized management structure and ministerial support for innovative educational practices, increased the perceived feasibility of implementing and maintaining these programs.

**Conclusions:**

This qualitative study of institution leaders and directors highlighted the challenges and facilitators to the deployment of an innovative continuous education model aimed at building capacity in the community for the management of various health conditions. Despite limitations, such as temporary initial funding, challenges in collecting performance indicators, most valued, and rigidity of the projects’ structure, results also show many characteristics (innovative model, alignment with the institution’s mission, and simplicity of its deployment) that helped move these projects toward sustainability within the institution. Results offer learning experiences that will be relevant to other settings evolving within a similar public health care system, wanting to implement this model.

## Introduction

Wait times to access specialized care in Canada are 242% longer than they were in 1993, now at about 12.6 weeks [1]. Rates of referrals to specialized care are influenced by many factors, ranging from practitioners’ degree of comfort and patient-doctor relationship to the occurrence of a global health crisis impacting referral pathways and delays, as demonstrated with the COVID-19 pandemic [2-5]. Given the rapidly evolving knowledge across the various fields of medicine and considering the limited capacity of specialized centers to absorb all of the patient referrals, it is essential to increase the capacity and comfort levels of primary care providers to manage increasingly complex cases.

Several innovative continuous education models have emerged, among which one is Project Extension for Community Healthcare Outcomes (ECHO) [6-8]. This continuous telementoring model aims to empower community-based health care professionals to provide care to individuals with specific conditions within their community. The model relies on videoconferencing meetings between hub experts and spokes (participants from the community) where there is a case discussion and didactic presentation. Four principles guide fidelity of the model when applied to new settings and new conditions: using technology to leverage scarce resources, using best practices to reduce disparity, using a case-based learning approach to better understand the complexity of patient presentation, and monitoring outcomes [9]. The benefits for health care providers of participating in such telementoring offerings in terms of knowledge, confidence, self-efficacy, and care delivery are well-established [6,10-15]. The model has been replicated and deployed in 202 countries (2813 programs) to address common and complex diseases [16]. Yet, there are known barriers to implementation and participation, including conflicting priorities and competing demands, issues of inclusiveness, time constraints, technology requirements, and a low degree of endorsement from the clinic leadership [17-23].

Beyond the characteristics of the intervention, from an organizational perspective, many inner and outer setting parameters, such as structural characteristics, population needs, local context, sources of funding, and external incentives, can influence the successful implementation of Project ECHO [24]. Little is known about systemic factors that must be considered when planning and implementing such a program and monitoring its growth within the institution, and how it might benefit more broadly the local health care ecosystem. Most implementation studies explored the postimplementation perspectives of health care professionals, whether participants (spokes) or hub experts, with little attention to date given to the perspectives of institutional leaders and managers.

In addition, many institutions harbor several ECHO programs within a single center [16]. However, data on the implementation processes of multiple programs within a single site are scarce. Agley and colleagues [25] explored the implementation of 5 programs within 1 site and found that Project ECHO is associated with change in practice and knowledge and reduced isolation of practitioners, but that rigorous studies of such a model are warranted.

The Consolidated Framework for Implementation Research (CFIR), created by Damschroder, comprising a set of 39 constructs spread across 5 domains, successfully guided the implementation of several projects across various health domains [26]. An adapted CFIR was developed by an ECHO implementation team to create ECHO-specific tools to help organizations assess their readiness and capacity to support an ECHO project and provide a checklist to support a successful implementation process [24].

The study objective was to explore, from an institutional and organizational standpoint, the systemic characteristics that influence the implementation of Project ECHO, their growth within an ecosystem, and their sustainability. Results will help guide other teams to successfully identify optimal implementation conditions for multiple programs within the same institution.

## Methods

### Study Setting

This study is part of a larger project aiming at evaluating the implementation of 3 Project ECHO programs (Hepatitis C, chronic pain, and concurrent mental health and substance use disorder) within a tertiary care, university-affiliated hospital center [27]. The pilot Project ECHO Centre hospitalier de l’Université de Montréal (CHUM) Hepatitis C program was launched in April 2017 to enable health care professionals to increase the rate of Hepatitis C screening, assessment, and treatment in Quebec remote areas. Shortly thereafter, the Project ECHO CHUM *Douleur Chronique* was launched in September 2017. Its goal is to foster knowledge regarding best practices in chronic pain management and enable collaboration between health care professionals of different degrees of specialization. Finally, a third program, Project ECHO CHUM Troubles Concomitants (concurrent mental health and substance use disorder), began in September 2018 to help health care professionals from different backgrounds gain knowledge in assessment, treatment, and referral of patients with concomitant mental health and substance use disorders.

### Study Design

This is a qualitative study comprised of focus groups and individual interviews conducted between November 2020 and April 2021 with stakeholders, directors, health care managers, project coordinators, and medical program leads within the hospital or the university network, and Project ECHO medical leads.

### Ethical Considerations

The study was approved by the research ethics committee of the CHUM (19.281). Written informed consent was obtained from all participants, and they could opt out from the study at any time. Data were deidentified prior to analyses. Participants did not receive a compensation for taking part in this study. The description of the study methods followed the Consolidated Criteria for Reporting Qualitative Research (COREQ) guidelines [28].

### Participants

Potential participants were targeted by the research team based on their role within and outside of the institution, to cover all relevant directions that were involved with the project, including (1) medical leads, coordinators, and managers involved directly or indirectly with the implementation and delivery of Project ECHO; (2) directors and health care managers of various hospital services and programs involved with Project ECHO (communication services, professional services, etc); and (3) stakeholders within the larger university network (Réseau universitaire intégré de santé et de services sociaux de l’Université de Montréal). A total of 30 potential participants were thus approached. Of the total, 13 participants were excluded because they refused to participate (n=5) or did not respond to our invitation (n=8). The 17 study participants included are presented in Table 1, which illustrates the roles and responsibilities of each participant within Project ECHO. Project ECHO spokes and experts were interviewed separately, and these data have been presented elsewhere [27]. Descriptions of participants’ characteristics are presented in Table 1.

**Table 1.** Characteristics of study participants.

Participants’ characteristics	Frequency, n (%)
Role	
ECHO^a^ medical leads	3 (18)
Project ECHO coordinators	4 (23)
Health care managers	2 (12)
Directors (teaching, academics, communication, and operations) and stakeholders	8 (47)
Gender	
Women	13 (77)
Men	4 (23)
Age (y)	
25‐34	1 (6)
35‐44	5 (29)
45‐54	3 (18)
55‐64	7 (41)
65+	1 (6)
Number of years involved in Project ECHO	
1	3 (18)
2	2 (12)
3	12 (70)
Employer	
Centre hospitalier de l’Université de Montréal	13 (77)
Réseau universitaire intégré de santé et de services sociaux de l’Université de Montréal (Integrated University Network for Health and Social Services at Université de Montréal)	4 (23)

a ECHO: Extension for Community Healthcare Outcomes.

### Recruitment

Potential participants were identified by the study investigators and those involved in the deployment of each of the 3 studied Project ECHO programs. Potential participants received an email inviting them to participate in a focus group. They were asked to express their interest by replying to the email, after which a research assistant explained the study procedure to them and scheduled the focus group. Those interested but unable to take part in a focus group because of scheduling conflicts were invited to participate in an individual interview. They were informed of the study’s objectives, namely to understand the barriers and facilitators to the deployment of ECHO programs and their sustainability, but they were not informed of the interviewers’ reasons or interests in the research topic.

### Procedure

Before the scheduled interview, participants received a link through email to complete an online consent form and a brief sociodemographic questionnaire. In total, 3 semistructured focus groups (2 with medical leads, project coordinators, and health care managers and 1 with directors and stakeholders) and 4 semistructured individual interviews were conducted online using the secured videoconferencing platform Zoom (Zoom Communications) by 1 of 2 women research team members (MGP [PhD] or ÉD [MSc]) trained and experienced (5+ y) in qualitative methods and were audio-recorded. Both interviewers had been working at the institution where the study was conducted for more than 5 years and knew many of the study participants in the context of other research projects or collaborations. Semistructured interview guides were built with open-ended questions aimed to explore topics such as barriers and facilitators to program implementation, interest toward this program, program fit within the larger institution and provincial health care ecosystem, and sustainability (Multimedia Appendix 1). Audio recordings were transcribed verbatim (transcripts were not returned to participants for comments due to time constraints). Field notes were taken during the interviews and used to supplement the data analysis. Focus groups lasted between 55 and 59 minutes (median 58 min) and varied in size between 3 and 6 participants, while individual interviews lasted between 18 and 28 minutes (median 24 min). Interviews were carried out in French, and the selected quotes were translated into English using a forward-backward translation process.

### Data Analysis

A qualitative content analysis with both deductive and inductive coding was used [29,30]. A deductive approach was used as a theoretical lens through which the data were interpreted [31], with initial domains mapping onto the Organizational Readiness and Implementation Tools for Project ECHO [24] (an adaptation of the CFIR [26]). These domains represented an initial starting point to understand the data, but other domains could be added to the framework as needed. More specifically, 1 analyst (MGP) read the transcripts many times and identified units of meaning that mapped into the participants’ perceptions of implementation barriers and facilitators of the projects within the institution. A coding sheet identifying the dimensions of the CFIR framework that fit the project’s context (acceptability, appropriateness, cost and trialability, and feasibility) was collaboratively identified with team members (ÉD and CW-B). Within each of these dimensions, inductive coding was used to identify subcategories and units of meaning. This step was performed by GP with frequent meetings with other analysts (ÉD and CW-B). When disagreements arose regarding data interpretation, we examined whether they stemmed from differences in backgrounds and sought to integrate these viewpoints to enrich the analysis. Memos were written throughout the analysis process. NVivo software (Lumivero) [32] was used to conduct the analyses. Participants’ feedback on the study results was not solicited.

### Reflexivity Statement

Members of the research team were involved in the development of ECHO programs at the institution (CW-B) or in the coordination of the research project (AT, RK, ÉD, and MGP). They come from the disciplines of medicine (AT and CW-B), social science (ÉD), environmental and occupational health (RK), and psychology (MGP). Team members directly involved in the execution of the research project and analysis (MGP and ÉD) engaged in reflexivity throughout the project, but most intensely during the elaboration of the study material (ie, development of interview guide), throughout data collection, and during the analysis, with the intent of reflecting on how our perspectives and experiences within the institution but also with regards to the research topic, influenced the orientation of the project, our understanding of the data, and how it shaped the findings. Reflexivity involved individual reflections through memos and note-taking as well as collaborative meetings.

## Results

### Overview

We generated themes based on available data pertaining to Project ECHO’s acceptability, appropriateness, cost and trialability, and feasibility (refer to Textbox 1 for a summary of the themes).

Textbox 1. **Summary of domains and themes.**

**Acceptability**

- Knowledge transfer as a core value in line with the institution’s academic and public health mission
- Innovation as an institutional pillar
- Institutional benefits—optimizing the patient flow in a 2-way stream

**Appropriateness**

- Versatility and structure of the ECHO (Extension for Community Healthcare Outcomes) model
- Meeting a growing need for education on rapidly evolving complex health conditions
- Rapid growth of ECHO programs

**Cost and trialability**

- Institutional sources of funding and human resources that benefit the outside community
- Lack of an easily measurable performance indicator
- Lack of recognition for time and resources invested

**Feasibility**

- Decentralized management structure
- Ministerial and academic characteristics of the outer setting bolstering the institution’s innovative educational practices

### Acceptability

The perceived acceptability of the Project ECHO programs was influenced by cultural aspects specific to the institution and its role within the larger health care network. The structure of this innovative program also contributed to increasing its acceptability.

#### Knowledge Transfer as a Core Value in Line With the Institution’s Academic and Public Health Mission

The institutional support for Project ECHO stemmed in part from the program’s fit with the institution’s academic mission. Being a university health network institution, its mandate beyond patient care involves knowledge transfer and supporting the wider health care community. From that perspective, Project ECHO uses an innovative continuous education model that enables expertise within the university health network and academic workforce to be shared with the broader medical community, which facilitates the execution of the institution’s academic mission as a center of expertise.

*I’d say that we have a great deal of specific expertise at the [institution], and our challenge is to share this expertise with partners who could benefit from it. Projects ECHO* *are in line with this and at the [institution], we try to generate knowledge, and from that knowledge we generated, how can we play our role as a member of a university health network within our network and how can we maybe support teams within this network. It builds pride in what they do, what they achieve, so I would say that there are not super demanding activities, not overly costly, but so worth it on multiple fronts. You cannot not support these initiatives and encourage them.*

[P4, Director]

#### Innovation as an Institutional Pillar

The Project ECHO distinguishes itself from other more conventional learning modalities, as it is based on ongoing real clinical cases and offers adaptability to have participants’ questions answered by a panel of clinical experts, using personalized solutions in an “all teach, all learn” approach. This leads to rich exchanges, making it possible for participants to have access to the experts’ reflections and decision-making processes that led to the proposed treatment path. Those components were identified as central to the success of such a learning model. Comments from managers and stakeholders underlined that one of the institutions’ characteristics that facilitated the deployment of those programs was the crucial importance given to innovative practices. This implied an agility to recognize innovative approaches and to maximize existing resources to bring them to life.

*However, to be an innovative institution, its also to see things differently, you know to have other ways of doing things. And so it was that, another way of looking at this and to say well, we have given a lot of training, of information, but telementoring, we had never done it. So, the openness that this brings…*

[P1, Director]

The emphasis put on innovative practices not only facilitated the implementation of the programs, but it also improved the likelihood of successful sustainability. Justifying the financial expenses and required resources for running Project ECHO was facilitated by a good comprehension of the project’s fit within the larger institutional academic mission and its core value of innovative practices.

#### Institutional Benefits-Optimizing the Patient Flow in a 2-Way Stream

Stakeholders described perceiving important institutional benefits from the Project ECHO programs in place. The projects were in line with the institution’s vision of patients receiving the right treatment at the right place at the right time. By helping health care providers within the broader health care system to care for their patients with complex health conditions, the institution contributed to facilitating optimal patient flow. This ensured that specialized services were used for the most complex cases, while less complex patients could access the right care in their community.

*Well, I think that it’s the main strength because otherwise our institution has 772 beds, not all of them are open, we cannot admit all of the patients so we must develop knowledge and expertise about specific clienteles and after that, help our network partners to care for these clienteles. With Projects ECHO* *, it helps do that. So, I would say that the strength of such programs is that it reinforces education, it reinforces partnership, it helps reinforcing multidisciplinary work and also the actors from different sectors.*

[P4, Director]

One way that Project ECHO facilitated this was through connecting and empowering first-line health care providers to treat more complex cases.

*We make sure that patients who are physically coming to the CHUM really need to be here, that they are the ones that fit with our mission. Because each time we accept a patient that could have been treated elsewhere, well we worsen access for another patient who probably must be seen at the CHUM. So I see a clear impact in terms of access, the famous “the right patient at the right place,” there really is an impact at that level. So for the CHUM, to allow the institution to recenter itself on its tertiary and quaternary mission, well it’s always a good thing that there are activities targeting primary and secondary care, we are after all a teaching institution.*

[P2, Director]

In addition, the experiences of clinical experts within the program benefited not only participants but the experts themselves. Through the constructive discussions taking place on complex cases, experts gained a better understanding of constraints other health care professionals are confronted with in their practice. These were perceived as factors enhancing the acceptability of the program by directors, managers, and stakeholders.

*Well those who do it, the team in fact, it helps them rethink, and then hearing, listening to what people working in primary and secondary care have to say, well it helps them too to discover other aspects that perhaps they might not have focused on previously. And I think that it brings the team closer because they to do that together. So even if these are people who work together, well in this case we can be a microbiologist who comes on board and the pharmacists is there… and that, that brings a lot.*

[P1, Director]

### Appropriateness

Appropriateness of Project ECHO fluctuated based on the structure of its delivery and on its fit with the outer setting.

#### Versatility and Structure of the ECHO Model

Project ECHO was perceived as attractive by managerial instances because of its operationalized structure that demonstrated effectiveness across various chronic health conditions and settings. This prestigious program’s history and the standardization of its delivery facilitated buy-in from various instances as little investment was needed to deploy the programs.

*Also, it’s a proven program, so it’s not just us, it’s elsewhere in the world… in Canada, in the United States, it’s well implemented. So, it’s had already proven its worth before we imported it here. That’s an important aspect.*

[FG7, Director]

On the other hand, the rigidity around the delivery of Project ECHO turned out in some instances to be an obstacle to its spread within the institution. This was either because it was perceived as less suitable for some pathologies since associated with higher costs than other telementoring alternatives, or because it lacked flexibility to use other technological platforms approved by ministerial instances.

*I don’t know if the ministerial position about the use of TEAMS, with for example teleconsultations, if that can be an obstacle. Well yes and no, maybe not. But if we would arrive at the conclusion that the two technologies do exactly the same thing, but with ECHO* *we must work with Zoom… I’ll give you an example. We are involved in the First Nations dossier. If we would realize that using Zoom for Project ECHO*^*®*^ *is technologically not feasible in this setting… well I think it would be a strong enough justification to use another model. I mean, the first priority, is to meet the needs. Then, if we can answer those needs through a standardized approach, it accelerates things, its more efficient in terms of deployment, maintenance, putting resources in common, etc. But it must meet the needs, otherwise we must find something else that will.*

[P2, Director]

#### Meeting a Growing Need for Education on Rapidly Evolving Complex Health Conditions

Successful implementation of the program depended in part on the adequacy between what was being offered and the need for such services within the broader medical community. In recent years, the increased complexity observed in various patient populations, the rapid evolution of therapeutic approaches, and the lack of educational resources targeting multiple concurrent diseases appeared to positively impact enrollment of participants in the program.

*For us, we have a large number of participants, but I think it answered a need, that people were a bit lost, people were helpless to know what to do with this clientele that is complex. So, I think that when they saw this, they jumped on it because there were no other services that provided this… It’s really difficult to be able to solve the problems this clientele has. So, to know that in one place, at the same time, many professionals from different disciplines. I think it was very tempting to have enrollees.*

[FG6, Coordinator]

By palliating gaps in the health care system and facilitating patient care within the network, this program was also perceived as indirectly benefiting the institution. This was perceived as one of the main advantages of Project ECHO from stakeholders’ perspectives.

*It is part of our mandate, so the admission to the institution, the return back to the network, all of that is part of the big network umbrella. So, it’s a bit for that reason that I was interested in Projects ECHO, because for us, obviously, we aim to optimize healthcare trajectory of patients who must come to our center or return to the network… So it is in this context that Projects ECHO* *are really interesting for our mission, because we have realized, us, while working with many clinical teams, that very often, a medical care is offered in our institution to compensate for a discomfort from certain partners in the network. And also, there are other ways of responding to this than having patients come here. So that’s how Projects ECHO* *fall in part within our institutional mission.*

[P3, Director]

Project ECHO was viewed as a valuable addition to the institutional service offerings because it had the potential to reach individuals beyond the actual participants and clinical experts involved in these sessions. In fact, the knowledge sharing that occurred between participants and their peers, as well as with other tertiary care centers, was significant. Several health care managers and stakeholders noted the snowball effects associated with the visibility of Project ECHO and the improvement of clinical practices within the network.

*So, to have a platform where when we do an intervention, we reach well 50, 70 other professionals, but in fact we maybe reach much more, because when the social worker from the team of first psychotic episodes in a clinic, often this person will talk to others during team meetings of 20 other professionals, and all of that. So, we reach a very large proportion of people.*

[FG5, Medical lead]

#### Rapid Growth of ECHO Programs

There has been a rapid growth within all 3 Project ECHOs over time. All 3 programs were implemented by individual medical leaders within an 18-month period with a rate of participation rapidly increasing over time. This rapid growth could be accommodated by the institution from a technology support standpoint. This expansion raised both awareness and interest within the institution and also within the broader medical community and other provincial health care networks.

*We always say that the CHUM is there for all individuals in Quebec, so ECHO is one way to help people from outside… The idea is to know, if you tell me tomorrow morning “we will go from 3 to 10 [programs],” well then we would need to assess the technical needs, how many times per week [technicians] need to be there, what this represents so that we can do this work together, so that it is a collaboration that works well. Because the idea is to support but we must plan well.*

[P3, Director]

### Cost and Trialability

Alongside the elements that impact acceptability and appropriateness of the programs, the absence of a long-term source of funding and the lack of easily measurable performance indicators remained important barriers to further program expansion and long-term sustainability of the existing programs.

#### Institutional Sources of Funding and Human Resources That Benefit the Outside Community

Basic costs of these programs involve access to telediffusion material such as webcams, screens, and computers, along with an available technology staff to troubleshoot any problems that can arise during live sessions. Considering the material and conference room being shared by the 3 programs, investments were optimized over the years.

Unlike other programs elsewhere in the country or in other countries, there was minimal funding from public sources to set up these 3 projects within the institution. The in-kind services offered by the institution, such as access to technology material and services, were made possible because of the fit between Project ECHO and the institutional mission. However, the absence of a permanent program coordinator position and of a dedicated paid medical lead posed a threat to the sustainability of the projects.

*In the United States, they often finance it, but in Toronto as well, they have a pretty big team compared to what we had. In terms of researchers, there are two doctors who are paid to do only ECHO* *with one salary. So, there is a really big paradigm between what is being asked here. And the risk is that it gets off track, and then stops, or the quality decreases, meaning that I can make recommendations in 15 minutes, but it won’t be of the same quality as if I were taking longer to write them. So, there is that organizational, financial aspect.*

[FG5, Medical lead]

Despite the recognized adequation between Project ECHO and the institutional mission, the lack of a permanent source of funding was identified as a constant threat to their survival.

*And let me tell you, I consider that it is not going fast enough, we should have more [programs], but we always hit the same problem when we say “I will need a budget to do that.”*

[P1, Director]

This instability might be due in part to the dual mission of the project, which is at the crossroads between educational and health care mandates. This overlap can generate confusion and a lack of accountability among funding bodies, who may assume that the program falls under the responsibility and the funding of other authorities.

*You know, its complex Project ECHO* *. There are multiple aspects. But at some level, it’s clear that after the meeting, at least from what I understood, there is an intention to help a patient from the start, which motivates, there is a discussion of real cases that will influence medical acts eventually. But in addition, in parallel, there is knowledge that people acquire more and more to become independent. So, there are these two aspects. One is clinical and one integrates knowledge transfer. And that, this aspect, is really novel.*

[FG7, Director]

#### Lack of an Easily Measurable Performance Indicator

Project ECHO programs are innovative educational models that benefit health care professionals within the community and patients. Currently, there are no easily accessible measurement metrics or dedicated resources to collect performance outcomes within the network. This poses a barrier to the long-term sustainability of the program. For example, it is difficult to document the exact number of patients that benefited from recommendations made within the ECHO sessions, or to estimate the number of patients who were not referred to specialized centers because of the knowledge and expertise gained by their primary health care provider. Given the very low budget allocated to Project ECHO, it became difficult for individual projects to collect empirical data to demonstrate their effectiveness. Because the data are limited, providing a strong rationale to maintain and expand the ECHO model within the institution was challenging. Such an issue is being recognized and must be addressed.

*We are going to have to think about how we will measure outputs from this process… and what is reasonable to invest considering the population gains in the end, but also for the knowledge development of our professionals. And… we cannot forget our professionals, our researchers, our experts, who learn a lot from people in the different regions, and not to remain disconnected from reality, it is important that communication goes in both directions. So, this has a worth. In my opinion, we probably do not measure it enough, and we probably don’t have enough indicators from a managerial standpoint to advertise the program to the level of the real impact it has for the population and for clinicians.*

[FG7, Manager]

#### Lack of Recognition for Time and Resources Invested

Despite the enthusiasm reported above, the time required to set up Project ECHO programs and maintain them reduced its acceptability. Such programs require leadership and skills to offer a high-quality experience. The tasks are often complex, including within and outside of the institution, to finalize the program logistics, create a cohesive hub team, recruit participants from the community, and carry out direct and indirect tasks related to the program. There was often a lack of recognition for the efforts invested. Beyond the actual time spent in an ECHO session, there was significant legwork required behind the scenes, which was not recognized financially.

*But Projects ECHO* *raise a lot of small questions, that are not evident from the start. And people think we put on a show you know, that it’s fun to do that, … but there is the visible side, but also the invisible side of ECHO. You know, there is the show, but after that, well you have to take care of it after. So it’s not that simple, when there is an investment from so many people. I think there are a lot of people who work for nothing in this program. We always work more than what we get paid for I think. And, that’s a reality for people who innovate, I think not necessarily the responsibility, but the impact that it has on each person. So, that’s what innovation looks like in [region], we never receive anything for the efforts that we put in.*

[FG5, Coordinator]

### Feasibility

Feasibility was enhanced by the managerial structure in place and also by the ministerial and academic characteristics of the outer setting that supported the institution’s innovative educational practices.

#### Decentralized Management Structure

The institution’s managerial structure is to assign a manager for each group of clientele, favoring, as a result, proximity management. This was identified as an organizational characteristic of the inner setting that facilitated the deployment of the Project ECHO model but also made it more difficult.

On the one hand, the decentralized management structure allowed each service to provide authorization for their staff to free up clinical and medico-administrative time to participate in the program, without needing approval from higher-level managers.

*Yes, the decentralized management structure in the patient populations I think is favorable because it is medical co-management and medico-administrative and proximity management so near sectors of care.*

[P4, Director]

On the other hand, however, decentralization increased the reliance on medical leaders to ensure the deployment and maintenance of programs over time. Project ECHO relied on the leadership of specific clinicians within each of the programs to set them up, mobilize their clinical teams, and obtain support from managers and stakeholders to access resources and disseminate the program within and outside of the institution. While there was an overwhelming institutional buy-in, the concrete steps to make this happen relied solely on those medical leaders.

For many participants, the existing structure, which provided a high level of autonomy within each clinical service, was a barrier to adequate communication within different levels of management about Project ECHO being put in place. Each of the 3 programs was developed through individual leaders’ initiatives, and as such, they were less visible within the hospital’s ecosystem. This led to suboptimal sharing of resources that could have accelerated the implementation of each program, had an institutional vision been in place earlier. For example, not all of the managers interviewed were aware of the different programs operating within the institution more than 3 years after their launch.

*I don’t know to what extent the Projects ECHO* *s are known from the larger CHUM community. Me I know them because they operate in sectors within my responsibility so we discuss them regularly. Do we hear about them more broadly, is everyone aware that we have initiatives such as Projects ECHO in other sectors? I have the impression that it’s less known and can be a barrier to further deployment of these programs.*

[P4, Director]

#### Ministerial and Academic Characteristics of the Outer Setting Bolstering the Institution’s Innovative Educational Practices

The institution was the first in the province to implement Project ECHO programs, so that their success depended on the support received at the ministerial and the integrated university network for health and social services (Réseau universitaire intégré de santé et de services sociaux de l’Université de Montréal [RUISSS]) levels. The RUISSS mission is to federate the university and its affiliated health and social services institutions to facilitate collaborations and set up special projects in line with its ministerial education and health mission. Project ECHO, being at the intersection of teaching and clinical practices, was a perfect fit with this mission. While the process took a few months to target the appropriate type and level of resources needed to implement the first program and required initial funding from pharmaceutical sponsors, the subsequent Project ECHO programs deployed benefited from the already established corridor.

*So there has really been a year and a half of processes, finding a key person at the RUISSS level has been key, because that person believed in the project from the start and helped me and the manager working there, who knew that person well, to meet the dean of pharmacy, make those connections for us…*

[FG5, Medical lead]

This collaboration between the RUISSS and the institution, early in the implementation phase, helped increase visibility of the projects within the medical community, which facilitated recruitment of participants and also increased awareness at the ministerial level of this type of program. The notoriety of Project ECHO worldwide, and the fact that the institution was the first university academic center to offer Project ECHO in French, highlights and contributes to the institution’s and the province’s visibility in innovative practices. Such progress could potentially lead to additional deployment of projects within the institution and within the province and play a role at the international level with the deployment of French-speaking Project ECHO programs.

*Let’s not forget that if we look at the strategic planning from the ministry for 2019-2023, there is an item about access to specialized care. But this type of ECHO, it’s exactly about making a specialty accessible in regions. So it could fall under that mandate…. Because it has been a while since we went to the ministry about that. But let’s not forget also that there is some pride. These are the first francophone ECHOs. We can rely on that. We can be francophone leaders for ECHOs.*

[FG7, Director]

## Discussion

### Overview

This study highlighted the rarely reported perspectives of executives and institutional partners, shedding light on the organizational components that are essential to the implementation and sustainability of Project ECHO. Results reflect the intricate balance between institutional resources and its broader mission within a provincial, public health care system. In terms of acceptability, the fit between the projects and the institution’s values of innovation, contribution to the broader community, and improving patient trajectory was central from the organizational leaders’ standpoint. The structure of the projects and their rapid growth within the institution confirmed an adequate fit with the organization. Initially, the projects benefited from temporary funds, but the absence of easily measurable performance indicators and a lack of recognition for clinicians’ invested time posed barriers to progress toward sustainability. Organizational characteristics, including a decentralized management structure and ministerial support for innovative educational practices, increased the perceived feasibility of implementing and maintaining these programs.

These results are in coherence with Mintzberg’s *Professional Bureaucracy* structural configuration of the institution under study [33,34], which is characterized by a strong focus on professionals placed as the operating core within the organization, standardization of skills to coordinate the different mechanisms, and the use of both vertical and horizontal decentralization so that professionals can benefit from a high degree of autonomy. Goals of such a professional bureaucracy structure are typically to innovate and provide high-quality health care services within a large, complex yet stable environment [33,34]. These key characteristics of the organization’s structure facilitate but also hinder the deployment and sustainability of initiatives like Project ECHO.

### Principal Results

The 3 initial Project ECHO programs launched at the institution were initiated by local experts who aimed to meet various needs, including building a stronger primary care network to reduce referrals to their specialized clinics and contribute to knowledge diffusion. In this context, the organization had little involvement in the initial setup of these programs, but its endorsement was essential to the sustainability and growth of the ECHO model within the institution. This is worrisome considering that a recent survey showed that across more than 1000 Project ECHO programs in 68 different countries, funding was temporary at the start [35]. The public health mission of this tertiary and quaternary care center and the potential disruptive nature and value creation associated with these projects were central to obtaining this endorsement, which enabled the launch of 3 additional Project ECHO programs since the completion of this study.

Considering Mintzberg’s organizational structures [33,34] can help understand how the inner setting (structural characteristics, culture, and mission alignment) and outer setting (financing, local conditions, and external pressure) characteristics highlighted in the CFIR framework can guide the development of an implementation strategy for Project ECHO. In this case, for example, professionals’ autonomy has been central to setting up the first operational year for each of these 3 projects, with little direct involvement from higher-level governance. This might not be possible in all organizations, especially if the organizational structure is less focused on professional autonomy, in which case the initial implementation strategy might need to include more rapid institutional leads.

Coherent with the professional bureaucracy organization structure, the executives also highly valued innovation in all of its forms. Project ECHO represented new ways within the province to train clinicians that showed promising results for both the recipients (primary care providers) and the deliverers (hub experts) and the institution. The benefits perceived by the participants of the 3 projects have been previously reported [27]. These bidirectional benefits were highly valued and helped the organization set up an infrastructure that would be able to maintain some growth of the innovation. These findings are similar to those reported in Australia, where executives highlighted key elements of Project ECHO that facilitated its deployment, including the alignment with the institution’s strategic priorities, innovative potential, and potential for integrated care [36].

The Project ECHO programs are highly standardized, including how sessions are carried out and the telehealth services needed for the sessions (Zoom). This had significant advantages as it minimized the program development required to launch a program, keeping it simple to generate the didactic content specific to each project. Conversely, some of these standardized procedures were incompatible with governmental commendations for the platforms to use for telehealth, which did not include Zoom. At the same time, it was possible for the 3 projects to share the room and licenses to deliver the sessions.

Even after the pandemic, Canada remains largely behind Commonwealth countries in terms of scheduling appointments online (22% in 2019 and 38% in 2022 in Canada compared with 56% in 2019 and 57% in 2022 in the Commonwealth countries), but the use of technology has been improving, particularly for the use of electronic medical records which increased by 20% from 2019 to 2022 [37]. It is likely that the initial technological barriers discussed in this study during the implementation of the ECHO programs before the pandemic have widely decreased by now.

The complexity of the health care ecosystem in the province made it difficult to collect performance indicators that are directly associated with health care performance. For example, the institution executives would like to understand how many patients are not referred to the institution because they received care from their provider locally. However, not all participants in the program would refer to this institution, as they might be living in a geographical region that is under the authority of a different tertiary care center. This lack of a direct performance indicator represents an important barrier to the sustainability of these programs. This difficulty in assessing the impact of the model on a population level is well-recognized [38]. A recent scoping review of patient and community health outcomes associated with Project ECHO showed that out of the 15 studies included, only 1 provided data on outcomes changed at the community level [38].

Finally, results highlighted some possible barriers to sustainability, namely, regarding the availability of human resources, such as dedicated time and funding for hub members to participate in sessions and write recommendations, as well as funding to cover operational costs. A 2023 review of financial structures of North American Project ECHO programs highlights that while many programs initially start up using institutional funds, those are rarely renewed in time, and finding permanent sources of funding becomes crucial [39]. There is a diversity of successful strategies that have been put in place, including aligning the program with state, provincial, or federal health priorities or finding external partners (eg, practice-based networks and translational institutes) [39].

### Limitations

This study provides the unique perspectives of organizational executives and leads as their institution implemented 3 different Project ECHO programs. Results are relevant to those institutions operating within public health care systems and want to implement and maintain these programs. Notwithstanding, this study has limitations. Results were collected in a single institution, and it would be important to validate these findings in other provinces and countries with a similar health care structure. Interviews took place after the implementation of the 3 projects, and thus, there might be a recall bias around questions pertaining to the early phases of implementation.

### Conclusions

This qualitative study, focusing on institution leaders and directors, highlighted the challenges and facilitators to the deployment of ECHO projects aiming at building capacity in the community for the management of various health conditions. Despite temporary initial funding, challenges in collecting performance indicators most valued, and perceived rigidity of the projects’ structure, our results show many characteristics (innovative model, alignment with the institution’s mission, and simplicity of its deployment) that helped move these projects toward sustainability within the institution. These learning experiences will be relevant to other institutions evolving within a similar public health care system, wishing to implement this model.

This study focused on the identification of factors at various levels that can influence the readiness for implementation but also the sustainability of an innovation and used the CFIR as a determinant framework for this purpose [40]. The CFIR is less adapted, however, to the collection of quantitative measures of implementation success [41]. It might be helpful for future studies to combine this model with other models, such as Proctor’s taxonomy of outcomes, to also document implementation outcomes.

## Supplementary material

10.2196/75844 Multimedia Appendix 1 Semistructured interview guide.

## Abbreviations

CFIR: Consolidated Framework for Implementation Research
CHUM: Centre hospitalier de l’Université de Montréal
ECHO: Extension for Community Healthcare Outcomes
RUISSS: Réseau universitaire intégré de santé et de services sociaux de l’Université de Montréal

## References

[1] Moir M, Barua B (2022). Waiting Your Turn: Wait Times for Health Care in Canada.

[2] Sperling S, Andretta C de L, Basso J (2022). Telehealth for supporting referrals to specialized care during COVID-19. Telemed J E Health.

[3] Burton C, Bajpai R, Mason KJ (2023). The impact of the COVID-19 pandemic on referrals to musculoskeletal services from primary care and subsequent incidence of inflammatory rheumatic musculoskeletal disease: an observational study. Rheumatol Adv Pract.

[4] Soltani SA, Fallah M, Marvi A (2024). Performance trend of the family physician referral system before and during the COVID-19 pandemic: a study in northern Iran. BMC Public Health.

[5] Tzartzas K, Oberhauser PN, Marion-Veyron R, Bourquin C, Senn N, Stiefel F (2019). General practitioners referring patients to specialists in tertiary healthcare: a qualitative study. BMC Fam Pract.

[6] Zhou C, Crawford A, Serhal E, Kurdyak P, Sockalingam S (2016). The impact of Project ECHO on participant and patient outcomes: a systematic review. Acad Med.

[7] Socolovsky C, Masi C, Hamlish T (2013). Evaluating the role of key learning theories in ECHO: a telehealth educational program for primary care providers. Prog Community Health Partnersh.

[8] Katzman JG, Galloway K, Olivas C (2016). Expanding health care access through education: dissemination and implementation of the ECHO model. Mil Med.

[9] Katzman JG, Comerci G, Boyle JF (2014). Innovative telementoring for pain management: project ECHO pain. J Contin Educ Health Prof.

[10] McBain RK, Sousa JL, Rose AJ (2019). Impact of Project ECHO models of medical tele-education: a systematic review. J Gen Intern Med.

[11] Anderson D, Zlateva I, Davis B (2017). Improving pain care with Project ECHO in community health centers. Pain Med.

[12] Mazurek MO, Parker RA, Chan J, Kuhlthau K, Sohl K, ECHO Autism Collaborative (2020). Effectiveness of the extension for community health outcomes model as applied to primary care for autism: a partial stepped-wedge randomized clinical trial. JAMA Pediatr.

[13] Mehrotra K, Chand P, Bandawar M (2018). Effectiveness of NIMHANS ECHO blended tele-mentoring model on integrated mental health and addiction for counsellors in rural and underserved districts of Chhattisgarh, India. Asian J Psychiatr.

[14] Nhung LH, Kien VD, Lan NP, Cuong PV, Thanh PQ, Dien TM (2021). Feasibility, acceptability, and sustainability of Project ECHO to expand capacity for pediatricians in Vietnam. BMC Health Serv Res.

[15] Ball S, Stryczek K, Stevenson L (2020). A qualitative evaluation of the pain management VA-ECHO program using the RE-AIM framework: the participant’s perspective. Front Public Health.

[16] Arora S (2024). Project ECHO 2024 annual report.

[17] Walters SM, Li WP, Saifi R (2022). Barriers and facilitators to implementing Project ECHO in Malaysia during the COVID-19 pandemic. J Int Assoc Provid AIDS Care.

[18] Becevic M, Smith E, Golzy M (2021). Melanoma extension for community healthcare outcomes: a feasibility study of melanoma screening implementation in primary care settings. Cureus.

[19] Shea CM, Gertner AK, Green SL (2021). Barriers and perceived usefulness of an ECHO intervention for office-based buprenorphine treatment for opioid use disorder in North Carolina: a qualitative study. Subst Abus.

[20] Salvador J, Bhatt S, Fowler R (2019). Engagement with Project ECHO to increase medication-assisted treatment in rural primary care. Psychiatr Serv.

[21] de la Garza Iga FJ, Mejía Alvarez M, Cockroft JD (2023). Using the Project ECHO™ model to teach mental health topics in rural Guatemala: an implementation science-guided evaluation. Int J Soc Psychiatry.

[22] Mubanga B, Fwoloshi S, Lwatula L (2023). Effects of the ECHO tele-mentoring program on HIV/TB service delivery in health facilities in Zambia: a mixed-methods, retrospective program evaluation. Hum Resour Health.

[23] Stevenson L, Ball S, Haverhals LM, Aron DC, Lowery J (2018). Evaluation of a national telemedicine initiative in the Veterans Health Administration: factors associated with successful implementation. J Telemed Telecare.

[24] Serhal E, Arena A, Sockalingam S, Mohri L, Crawford A (2018). Adapting the Consolidated Framework for Implementation Research to create organizational readiness and implementation tools for Project ECHO. J Contin Educ Health Prof.

[25] Agley J, Delong J, Janota A, Carson A, Roberts J, Maupome G (2021). Reflections on Project ECHO: qualitative findings from five different ECHO programs. Med Educ Online.

[26] Damschroder LJ, Aron DC, Keith RE, Kirsh SR, Alexander JA, Lowery JC (2009). Fostering implementation of health services research findings into practice: a consolidated framework for advancing implementation science. Implement Sci.

[27] Develay É, Wartelle-Bladou C, Talbot A (2024). Implementation of Project ECHO in a university health network: contrasting and comparing experiences across health conditions through a qualitative approach in a Canadian tertiary care centre. BMJ Open.

[28] Tong A, Sainsbury P, Craig J (2007). Consolidated criteria for reporting qualitative research (COREQ): a 32-item checklist for interviews and focus groups. Int J Qual Health Care.

[29] Elo S, Kyngäs H (2008). The qualitative content analysis process. J Adv Nurs.

[30] Elo S, Kääriäinen M, Kanste O, Pölkki T, Utriainen K, Kyngäs H (2014). Qualitative content analysis: a focus on trustworthiness. SAGE Open.

[31] Crabtree B, Miller W (1999). Doing Qualitative Research.

[32] (2017). QSR International.

[33] Lunenburg FC (2012). Organizational structure: Mintzberg’s framework. Int J Schol Acad Intellect Divers.

[34] Mintzberg H (1989). Mintzberg on Management Inside Our Strange World of Organizations.

[35] Moss P, Hartley N, Russell T (2024). Project ECHO^®^: a global cross-sectional examination of implementation success. BMC Health Serv Res.

[36] Moss P, Hartley N, Ziviani J, Newcomb D, Russell T (2020). Executive decision-making: piloting Project ECHO® to integrate care in Queensland. Int J Integr Care.

[37] (2023). Canadian Institute for Health Information, The Expansion of Virtual Care in Canada: New Data and Information.

[38] Osei-Twum JA, Wiles B, Killackey T, Mahood Q, Lalloo C, Stinson JN (2022). Impact of Project ECHO on patient and community health outcomes: a scoping review. Acad Med.

[39] Larson R, Day SK, Dodsworth-Rugani K (2023). PROJECT ECHO implementation: guidance from the field: frequently asked questions. https://www.researchgate.net/publication/369550690.

[40] Nilsen P (2015). Making sense of implementation theories, models and frameworks. Implement Sci.

[41] GBD 2021 Low Back Pain Collaborators (2023). Global, regional, and national burden of low back pain, 1990-2020, its attributable risk factors, and projections to 2050: a systematic analysis of the Global Burden of Disease Study 2021. Lancet Rheumatol.

