# Supplementary material for: Exploring the Implementation of Multiple Telementoring ECHO Programs From an Institutional and Organizational Perspective: Qualitative Study
Source: JMIR Med Educ. 2025 Nov 13;11:e75844. doi: 10.2196/75844 (PMC12614116; doi:10.2196/75844)
Supplement: Multimedia Appendix 1 [file mededu-v11-e75844-s001.docx]

Semi-structured Interview Guide

1. To start, I would like for you to explain your role within the projects ECHO at the CHUM
   1. In which ways were you involved during the implementation period
   2. How are you involved now
2. How were the projects ECHO initially perceived at the CHUM?
   1. In your opinion, what are the specific characteristics of the CHUM that made it possible to implement projects ECHO
   2. Why were these projects important
   3. How were these projects contribution to the various missions of the institution?
3. What are the strengths and difficulties associated with the projects ECHO?
   1. Barriers and facilitators associated with implementing and sustaining the projects
4. What were the challenges encountered during the implementation of the first project ECHO?
   1. How were these challenges addressed?
5. What were the challenges that persisted or arose as the subsequent projects were implemented?
   1. How were these challenges addressed?
6. What were the positive and negative repercussions of having implemented projects ECHO?
   1. In terms of care trajectories
   2. In terms of healthcare delivery
   3. In terms of expertise
   4. In terms of human resources
   5. At the institution level
7. Which elements or factors were necessary to sustain these projects
8. Why and how the CHUM became a leader at the provincial level by implementing projects ECHO?
   1. Regional challenges
   2. Provincial challenges
9. What suggestions do you have based on this experience so that the CHUM can continue implementing more projects ECHO?
